# Redox Signaling and Neural Control of Cardiovascular Function

**DOI:** 10.1155/2016/7086018

**Published:** 2016-03-30

**Authors:** Hanjun Wang, Adam J. Case, Wei-Zhong Wang, Patrick J. Mueller, Scott A. Smith

**Affiliations:** ^1^Department of Anesthesiology, University of Nebraska Medical Center, Omaha, NE 68198-4455, USA; ^2^Department of Cellular and Integrative Physiology, University of Nebraska Medical Center, Omaha, NE 68198-5850, USA; ^3^Department of Physiology, Second Military Medical University, 800 Xiangyin Road, Shanghai 200433, China; ^4^Department of Physiology, Wayne State University, 5263 Scott Hall, 540 E. Canfield, Detroit, MI 48201, USA; ^5^Departments of Health Care Sciences and Internal Medicine, UT Southwestern Medical Center, Dallas, TX 75390, USA

Redox signaling has been widely reported to be involved in modulation of the cardiovascular system in both healthy and disease conditions. Redox imbalance such as oxidative stress contributes to the pathological process of many cardiovascular-related diseases such as diabetes, heart failure, and hypertension while also playing a negative role in the aging process [1–4]. Overactivation of the sympathetic nervous system (SNS) is a key event in each of these conditions. However, it is not fully understood how redox signaling interacts with the SNS in either aging or cardiovascular diseases. In this special issue, a number of contributions have been made to address this issue. An original research article by E. Moya et al. demonstrated that peroxynitrite formation mediates chronic intermittent hypoxia-induced hypertension via carotid body chemosensory potentiation. This study provides an excellent example that redox signaling can activate the SNS via an interaction with peripheral chemosensory afferents. On the other hand, J. P. Collister and colleagues reported that selective overexpression of superoxide dismutase (SOD) in one of the circumventricular organs (organum vasculosum of the lamina terminalis, OVLT) via injection of adenoviral vectors encoding human CuZnSOD (SOD1) significantly decreases blood pressure in an AngII-induced rat model of hypertension. This finding suggests that redox signaling in the central nervous system (e.g., OVLT) might play an important role in the development of AngII-induced hypertension. Finally, J. Hatcher and colleagues used SOD1 transgenic mice to determine the effect of reactive oxygen species (ROS) on arterial baroreflex sensitivity. They demonstrate that global overexpression of SOD1 preserves normal blood pressure (BP) and heart rate (HR) but enhances aortic depressor nerve function in mice. Although the authors did not further address the issue in which part of baroreflex arc was affected by overexpression of SOD1, this study did provide strong evidence that redox signaling might be involved in autonomic regulation.

In addition to identification and description of central mechanisms mediated by redox signaling, understanding how sex differences also contribute to these physiological processes is becoming increasingly important. In this special issue, original research articles by F. Hao et al. and S. Dai et al. highlight that female sex hormones, such as estrogen, are critical to the operation of central neural pathways that modulate cardiovascular function through antioxidant and anti-inflammation mechanisms. The article by F. Hao et al. reports that estrogen replacement reduces oxidative stress in the rostral ventrolateral medulla (RVLM) as well as sympathetic outflow in ovariectomized (OVX) rats. These findings elucidate a potential antioxidant role of estrogen in the central nervous system. The second article by S. Dai et al. suggests that the chronic antihypertensive and anti-inflammatory effects of Compound 21 (a selective angiotensin type 2 receptor (AT2R) agonist) in DOCA/NaCl-induced female hypertensive rats may function through female sex hormones. As evidence, these beneficial effects of Compound 21 could be abolished by OVX.

Several papers in this special issue also described new insights into the antioxidant therapeutic potential of various pharmacological and dietary compounds in spinal cord injury, asthma, and cardiomyopathy. M. Su and colleagues demonstrated a role for histone deacetylase-6 (HDAC6) in the protection of neurons after spinal cord injury and the resulting hypoxia-ischemia insult. The investigators demonstrate that inhibition of HDAC6* in vivo* and* in vitro* exacerbates ROS production and neuronal apoptosis, indicating that HDAC6 might serve as a new antioxidant target in neurotrauma. In another report in this issue, Y. Ma and colleagues provided evidence that an active compound found in figs and mulberries, known as morin, abrogated immune cell migration as well as cytokine production and ROS attenuating inflammatory processes in a model of asthma. This study highlights a novel profile of morin as a potent antioxidant and anti-inflammatory agent for asthma. In addition, a paper by M. Zhao et al. investigated the potential redox signaling pathways involved in cardiomyocyte hypertrophy induced by palmitic acid, the most common saturated fatty acid found in animals, plants, and microorganisms. These investigators demonstrated that palmitic acid-induced cardiomyocyte hypertrophy is associated with activation of necroptosis markers such as receptor interacting protein kinases (RIPK) 1 and 3. Moreover, the authors observed crosstalk between ER stress and necroptosis induced by palmitic acid. They suggested that mammalian target of rapamycin (mTOR) signaling was a key factor mediating the palmitic acid-induced activation of necroptosis and cardiac hypertrophy. Finally, the article by T. Dong et al. provided a very interesting observation that renal denervation slowed the development of deep venous thrombosis (DVT). They utilized several different measures to show that the mechanisms related to DVT are related to oxidative stress, as antioxidants such as tempol (small molecule superoxide scavenger) could achieve a protective effect similar to renal denervation. These findings extend our understanding of how neurons may affect the circulation via interactions with redox signaling.

The final topic of this special issue is related to exercise. It is well known that acute exercise can dramatically increase ROS production whereas long-term exercise training (ExT) plays an antioxidant and anti-inflammatory role [5–7]. Two papers in this special issue are focused on the ExT-mediated cardiovascular benefits in hypertension and diabetes, respectively. C. Ren and colleagues examined the effects of ExT on the components of brain renin-angiotensin system in the RVLM of spontaneously hypertensive rats (SHR). The data indicated that ExT for 12 weeks decreased the expression of ACE and AT-1R in the RVLM but increased the expression of ACE-2 and Mas receptors. Therefore, the authors concluded that ExT improves hypertension via resetting the balance of ANG II and ANG 1–7 at the level of the RVLM. N. Sharma and colleagues reported that ExT significantly reduced expression of NADPH oxidase subunits p47 and p67 in hearts of streptozotocin-induced diabetic rats. They also confirm that ExT reduced the elevated levels of collagen type III in diabetic hearts. Taken together, these data suggest that ExT attenuates oxidative stress in the diabetic heart. This new evidence is useful to our understanding of the beneficial effects of ExT on the cardiac extracellular matrix, cardiac function, and cardiac remodeling in diabetes.

In summary, the articles presented in this special issue highlight the current advances in the research fields of redox biology, neural science, and exercise physiology. These articles enrich our understanding of how redox signaling interacts within the nervous system both at rest and during exercise. Moreover, the works highlight the therapeutic potential of antioxidants as effective treatments for various cardiovascular and neural diseases.
